# TWIST1, a gene associated with Saethre-Chotzen syndrome, regulates extraocular muscle organization in mouse

**DOI:** 10.1016/j.ydbio.2022.07.010

**Published:** 2022-08-06

**Authors:** Mary C. Whitman, Nicole M. Gilette, Jessica L. Bell, Seoyoung Kim, Max Tischfield, Elizabeth C. Engle

**Affiliations:** 1 Department of Ophthalmology, Boston Children’s Hospital, Boston, MA 02115; 2 Department of Ophthalmology, Harvard Medical School, Boston, MA 02115; 3 F. M. Kirby Neurobiology Center, Boston Children’s Hospital, Boston, MA 02115; 4 Department of Neurology, Boston Children’s Hospital, Boston, MA 02115; 5 Department of Neurology, Harvard Medical School, Boston, MA 02115; 6 Howard Hughes Medical Institute, Chevy Chase, MD

**Keywords:** Twist1, extraocular muscles, strabismus, tendons, cranial nerves, craniosynostosis

## Abstract

Heterozygous loss of function mutations in *TWIST1* cause Saethre-Chotzen syndrome, which is characterized by craniosynostosis, facial asymmetry, ptosis, strabismus, and distinctive ear appearance. Individuals with syndromic craniosynostosis have high rates of strabismus and ptosis, but the underlying pathology is unknown. Some individuals with syndromic craniosynostosis have been noted to have absence of individual extraocular muscles or abnormal insertions of the extraocular muscles on the globe. Using conditional knock-out alleles for *Twist1* in cranial mesenchyme, we test the hypothesis that *Twist1* is required for extraocular muscle organization and position, attachment to the globe, and/or innervation by the cranial nerves. We examined the extraocular muscles in conditional *Twist1* knock-out animals using *Twist2*-cre and *Pdgfrb*-cre drivers. Both are expressed in cranial mesoderm and neural crest. Conditional inactivation of *Twist1* using these drivers leads to disorganized extraocular muscles that cannot be reliably identified as specific muscles. Tendons do not form normally at the insertion and origin of these dysplastic muscles. Knock-out of *Twist1* expression in tendon precursors, using *scleraxis*-cre, however, does not alter EOM organization. Furthermore, developing motor neurons, which do not express *Twist1*, display abnormal axonal trajectories in the orbit in the presence of dysplastic extraocular muscles. Strabismus in individuals with *TWIST1* mutations may therefore be caused by abnormalities in extraocular muscle development and secondary abnormalities in innervation and tendon formation.

## Graphical Abstract

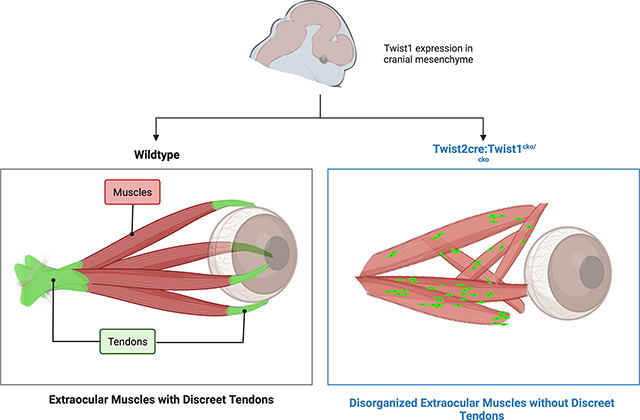

## Introduction

TWIST1 is a basic helix-loop-helix transcription factor required for the specification of cranial mesoderm (Qin et al., 2012). Heterozygous loss of function of *TWIST1* in humans causes Saethre-Chotzen syndrome, which is characterized by craniosynostosis, facial asymmetry, ptosis, strabismus, and distinctive ear appearance (Gallagher et al., 1993). Patients with craniosynostosis, especially syndromic craniosynostosis, have a very high incidence of V-pattern strabismus. Several potential mechanisms have been postulated, including malrotation/mispositioning of the orbits secondary to the craniosynostosis, absence of certain extraocular muscles (EOMs), or mispositioning of the EOMs on the globe (Rosenberg et al., 2013). Additionally, some individuals with *TWIST1* variants have isolated ptosis, without clinically apparent craniosynostosis (Dollfus et al., 2002; Stoler et al., 2009). We therefore sought to evaluate the role of *Twist1* in EOM differentiation and development.

The genetic pathways regulating extraocular muscle differentiation are distinct from those of somatic muscle and of adjacent craniofacial muscle (Buckingham and Rigby, 2014). In primordial EOM cells, PITX2 activates a myogenic regulatory factor (MRF) cascade involving MYF5 and MRF4, leading to the activation of MYOD, a myogenic determination factor. EOM precursors do not express PAX3 or PAX7, which activate MYOD expression in somitic muscle precursors (Sambasivan et al., 2009; Sambasivan et al., 2011). Thus, complete loss of either *Pitx2*, or combined loss of *Myf5* and *Mrf4*, results in an absence of EOMs (Diehl et al., 2006; Gage et al., 1999; Kitamura et al., 1999; Sambasivan et al., 2009). EOMs differentiate from cranial mesodermal precursors in periocular mesenchyme (Bothe and Dietrich, 2006; Fan et al., 2016; Kuroda et al., 2021; Ziermann et al., 2018). The muscles initially form an anlage in the orbit. Between E11.75 and E12.5, they separate into distinct EOMs which originate at the annulus of Zinn and insert on the sclera (Comai et al., 2020; Michalak et al., 2017). The connective tissues at the insertion of the muscles on the sclera derive from neural crest and mesodermal precursors while the connective tissues at the origin of the muscles at the annulus of Zinn derive from mesodermal precursors (Comai et al., 2020; Grimaldi and Tajbakhsh, 2021; Kuroda et al., 2021). The cell-intrinsic factors and extrinsic cues which regulate the growth and organization of the EOMs are not well characterized.

*Twist1* null mice die at mid gestation (~E10.5) and have complete failure of cranial neural fold fusion; chimeric mice with *Twist1* null cells in the cranial mesenchyme also have exencephaly (Chen and Behringer, 1995). Heterozygous mice have limb abnormalities and premature fusion of the cranial sutures (Bourgeois et al., 1998; Carver et al., 2002). Due to the severe consequences of full loss of *Twist1*, several studies have used conditional alleles with a variety of cell-type specific cre recombinase lines to examine the role of *Twist1* in various aspects of development (Bildsoe et al., 2009; Bildsoe et al., 2013). Loss of *Twist1* in the cranial neural crest, with *Wnt1*-cre, leads to severe malformations of the facial and skull bones, as well as abnormalities of eye development (Bildsoe et al., 2009). Loss of *Twist1* in cranial mesodermal progenitor cells via *Mesp1*-cre abolishes *Pitx2* expression in the periocular area and leads to complete loss of the EOMs, including the initial muscle anlage. However, these animals, like the complete null, have neural tube closure defects (Bildsoe et al., 2013). *Mesp1*-cre is expressed in mesoderm starting at gastrulation (Saga et al., 1999); conditional knock-out of *Twist1* under other mesenchymal cre drivers with later onset of expression allow neural tube closure and more complete head patterning (Tischfield et al., 2017).

To determine the role of *Twist1* in extraocular muscle development without the confounding effects of exencephaly, we have examined the extraocular muscles in mice with loss of *Twist1* in different subpopulations of mesenchyme and show that *Twist1* expression is required for proper EOM patterning and tendon formation. Furthermore, developing motor neurons, which do not express *Twist1*, display abnormal branching in the presence of dysplastic EOMs.

## Methods

### Animals

Mouse lines used were: *Twist1*^*cKO*^ (B6;129S7-*Twist1*^*tm2Bhr*^/Mmnc; MMRRC 016842-UNC) (Chen et al., 2007); *Twist2*-cre (B6.129X1-*Twist2tm1.1(cre)Dor*/J; Jackson Labs 008712) (Sosic et al., 2003); *Scx*-cre (Gift of Ronen Schweitzer; (Blitz et al., 2009)); *Scx*-GFP (Gift of Ronen Schweitzer; (Pryce et al., 2007)); *Isl*^MN^-GFP (Tg(Isl1-EGFP*)1Slp; Jackson Labs 017952) (Lewcock et al., 2007); *Pdgfrb*-cre (Cuttler et al., 2011). All animal work was approved and completed in compliance with Boston Children’s Hospital Institutional Animal Care and Use Committee protocols (20-01-4061R).

### Frozen Section Immunohistochemistry

Embryos were fixed in 4% paraformaldehyde for 2–24 hours depending on the age, washed briefly with PBS, and transferred to a solution of 30% sucrose in PBS until they sank. Embryos were then embedded in Neg-50 (Richard Allan Scientific, San Diego, CA), frozen on dry ice, and stored at −80°C until they were sectioned. Immunohistochemistry was done using 20-um frozen sections cut on a Leica CM3050 S cryostat (Buffalo Grove, IL, USA). Prior to staining, sections were equilibrated to room temperature for at least 30 minutes. Antigen retrieval was performed using a citrate-based buffer (Antigen Unmasking Solution H-3300; Vector Laboratories, Burlingame, CA, USA) and steam for 20 minutes. Sections were then blocked in 2% normal goat serum in PBS with 0.1% Triton X-100 (PBS-TritonX), incubated in primary antibody overnight at 4°C, washed 3 × 3 minutes with PBS-TritonX, incubated in secondary antibody for 1 hour at room temperature, washed 2 × 3 minutes with PBS-TritonX, stained with DAPI (1:10,000) for 3’ in PBS, washed one last time for 3 minutes with PBS, and mounted in Fluoromount-G mounting media (Thermo Fisher Scientific, Waltham, MA, USA). Images were captured on a Zeiss LSM 700 series laser scanning confocal microscope (Carl Zeiss Meditec, Oberkochen, Germany).

### Fluorescent Whole-Mount Embryo Immunohistochemistry

#### iDISCO

Whole-mount E13.5, 14.5, and E15.5 embryos were prepared using a modified version of the previously described iDISCO+ protocol (Renier et al., 2014; Whitman et al., 2019). Embryos were first collected and fixed overnight in 4% paraformaldehyde at 4°C, washed with PBS, dehydrated through a graded methanol series, bleached overnight in 5% H_2_O_2_ in methanol, rehydrated through a graded methanol series, and washed in PBS with 2% Triton-X. Embryos were then incubated at 37°C in permeabilization solution for 2 days, blocking solution for 2 days, and incubated in primary antibody diluted in PBS with 2% Tween-20 and heparin (PtWH), 5% DMSO, and 3% donkey serum for 7 days, washed with PtWH for one day at room temperature, and incubated with secondary antibody diluted in PtWH with 3% donkey serum at 37°C for 7 days. After staining, embryos were washed in PtWH, dehydrated through a graded methanol series, and incubated in a methanol and dichloromethane solution. Embryos were then cleared using dibenzyl ether (DBE). Embryos were mounted on coverslips in DBE and imaged using a Zeiss LSM 700 series laser scanning confocal microscope. Images were acquired with Zen Software (Carl Zeiss Micro-Imaging GmbH, Göttingen, Germany) and manipulated in three dimensions using Arivis software (Zeiss, Rostock, Germany). To facilitate 3D visualization of orbital structures at E15.5, arteries were selected using the find large objects feature and selectively cropped out of displayed images.

##### Quantification

Orbital Images were reconstructed in Arivis software (Zeiss, Rostock, Germany). EOMs were selected using the find large objects feature, and total volume of EOMs calculated. Graphs and statistical analysis (student’s t test) were done using GraphPad software.

### Cleared Orbital Sections

E12.5-E15.5 mouse embryos were collected and heads were fixed overnight in 4% paraformaldehyde at 4°C with orbital shaking. After a wash with phosphate buffered saline (PBS), embryo heads were fixed overnight in Dent’s fix (80% Methanol/20%DMSO). Samples then incubated with primary antibody diluted in PBST (0.5% TritonX-100, 10% NGS) for 2–5 days (age dependent) at 4°C followed by several washes in PBST. Final washes done with PBS. anti-SMA used for wholemount EOM preps is Cy-3 conjugated, mitigating the need for a secondary antibody incubation. Samples were dehydrated through a series of 1 hr methanol incubations of increasing concentration (25%, 50%, 75%, 100%) and then cleared for several days in glass vials filled with 1:2 benzyl alcohol:benzyl benzoate (BABB) and stored at RT until imaging. Samples oriented in a 3D cut imaging chamber sandwiched between two glass coverslips and submerged in BABB. Images of EOMs were collected with a Zeiss LSM700 laser scanning confocal microscope and a 10X, 0.45 NA ApoChromat objective. Visualization of the orbit and annulus defined limits of the z-stack. Post-image processing was performed with Zen (Zeiss).

### Antibodies

Mouse Anti-Actin, a-Smooth Muscle-Cy3 (1:750, RRID AB 476856; MilliporeSigma, Burlington, MA, USA), rabbit anti-GFP (1:500, RRID AB221569; Thermo Fisher Scientific, Boston, MA, USA), Mouse anti-MYOD (1:500, RRID AB 1098295; Thermo Fisher Scientific, Waltham, MA, USA), Mouse Anti-PITX2 (1:500, RRID AB 509266; Abnova, Taipei City, Taiwan), Alexa-Fluor 546 goat anti-mouse(1:1000, RRID AB 141370; Molecular Probes, Eugene, OR, USA), Alexa-Fluor 647 goat anti-rabbit (1:1000, RRID AB 141663; Invitrogen, Carlsbad, California, USA,), Alexa-Fluor 647 donkey anti-mouse (1:1000, RRID AB 162542; Molecular Probes, Eugene, OR, USA).

## Results

### Loss of *Twist1* in subpopulations of cranial mesenchyme results in disorganized EOMs.

To determine the role of *Twist1* expression on EOM precursor differentiation and development, we produced conditional knock-outs using mesenchymal cre drivers with later, more limited expression than *Mesp1*-cre. In these animals, the neural tube closes and head formation is grossly normal (Tischfield et al., 2017).

**Figure 1.**
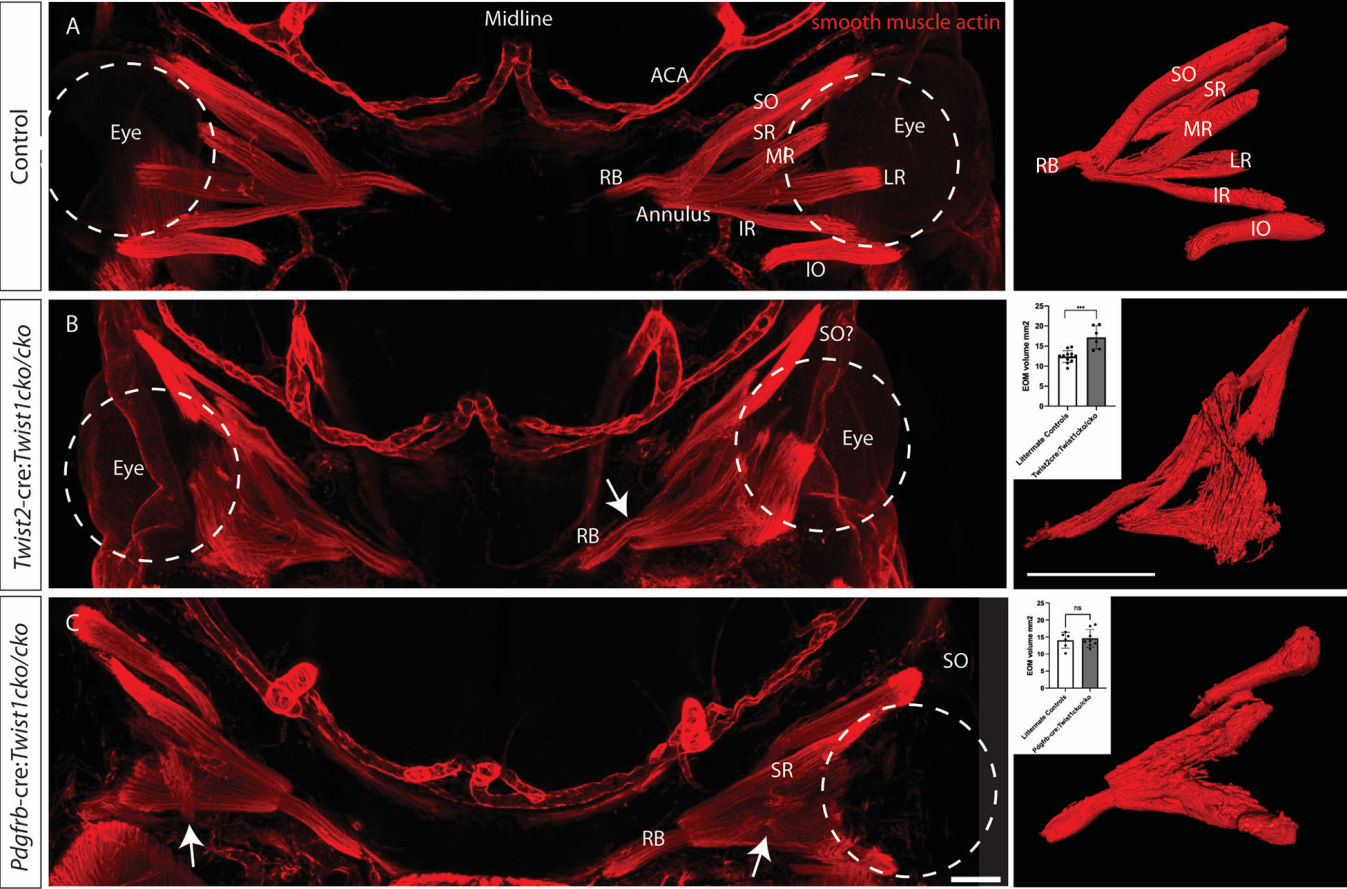
Loss of *Twist1* in different subsets of mesenchyme leads to varying degrees of EOM disorganization. Whole mounts from E13.5 embryos stained with anti-smooth muscle actin and imaged coronally show (A) the normal arrangement of the EOMs around the eye (dashed circle indicates left eye with red EOMs labeled) and the arteries of the anterior head. To the right of each image is a 3D reconstruction of the EOMs. (B) Loss of *Twist1* in *Twist2*-cre expressing cells leads to profound disorganization of the EOMs. There is a muscle superiorly that appears to make a turn as if going through the trochlea, which may be the superior oblique (labeled SO?). This muscle, however, appears different from the wild-type superior oblique, in which muscle tissue extends only to the trochlea, not through it (compare to SO in panel A; in wild-type mice, a tendon (not labeled) extends from the globe to the trochlea). The RB and annulus of Zinn can be identified (arrow), but the other EOMs do not separate into distinct muscles. The total volume of EOMs is greater in the mutant animals (graph, inset). (C) Loss of *Twist1* in *Pdgfrb*-cre expressing cells leads to mild disorganization of the EOMs. The SR, SO, and RB can be identified, but the other EOMs do not fully separate into distinct muscles, and there are muscle fibers oriented obliquely across the orbit, not originating at the apex (arrow). Consistent with the less severe phenotype, the total volume of the EOMs is not different from littermate controls (graph, inset). RB: retractor bulbi, IR: inferior rectus, MR: medial rectus, SR: superior rectus, LR: lateral rectus, SO: superior oblique, IO inferior oblique. ACA: anterior cerebral artery. Scale bar equals 200 um. n = 3–6 for each genotype. Control images are from littermate controls who do not express cre recombinase and/or have wildtype *Twist1* alleles.

We first examined conditional loss of *Twist1* with *Twist2-cre*. TWIST2 is a bHLH transcription factor with high homology to Twist1. Spatially, *Twist1* and *Twist2* have similar expression patterns in mesenchymal tissues, but temporally *Twist2* is expressed after *Twist1* (Franco et al., 2011). *Twist2* is expressed in medial cranial mesenchyme as early as E8.5 and E9.5 and is involved in maintenance of mesenchymal state (Bildsoe et al., 2016). *Twist2*-cre expression has been shown as early as E9.5 in mesenchymal tissues (He et al., 2017), Supplemental Figure 1). In these animals at E13.5, we found that the EOMs are present, but are severely disorganized compared to littermate controls that do not express cre and/or are wildtype for the *Twist1* allele (Figure 1A–B). The annulus can be identified but the extraocular muscle fibers do not all appear to originate from it. Moreover, individual muscles cannot be identified beyond a putative retractor bulbi muscle (RB), and a muscle that may be the superior oblique, as it appears to go through the trochlea. Muscle fibers are present at a variety of orientations within the orbit, and the insertions to the globe cannot be identified. The total muscle volume of the EOMs was quantified and the mutant animals showed an increase in total volume (17.16+/−2.89 mm^3^ in mutants, vs 12.37 +/− 1.48 in controls, p=0.0002). One day earlier, at E12.5, the individual muscles cannot be identified in the mutants (not shown).

To confirm our findings, we examined knock-out of *Twist1* with *Pdgfrb*-cre (Cuttler et al., 2011), a similar mesenchymal marker, which diffusely labels cells in the head, including neural crest and mesoderm-derived mesenchyme (Tischfield et al., 2017). These mice have a similar, although less severe, phenotype with disorganization of the EOMs (Figure 1C). The RB is positioned normally, and the superior rectus (SR) and superior oblique (SO) muscles also appear properly positioned. The medial rectus (MR), lateral rectus (LR) and inferior oblique (IO) muscles, however, do not separate properly into distinct muscles, and there are ectopic muscle fibers across the orbit (white arrows in Figure 1C). Consistent with the milder phenotype, the total volume of the EOMs was not different in mutant animals, compared to littermate controls (14.61 +/− 2.59 vs 14.03 +/− 2.32, p=0.67). Together, these findings indicate that expression of *Twist1* in mesenchyme is necessary for EOM organization.

To further elucidate the extent to which *Twist1* is required in mesenchymal tissues for EOM organization we focused the rest of our investigations on the animals with the more severe EOM organization phenotype, using the *Twist2*-cre driver.

### The abnormal muscle has normal PITX2 and MYOD expression

**Figure 2.**
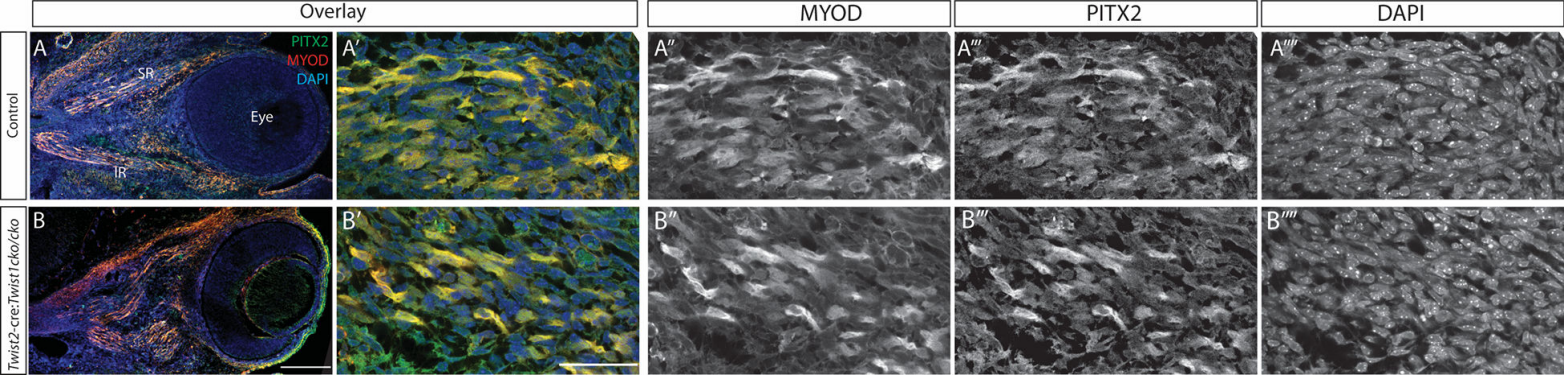
PITX2 and MYOD expression are normal in the disorganized EOMs. Sagittal frozen sections stained with anti-PITX2 antibody (green) and anti-MYOD antibody (red) show equal PITX2 and MYOD expression in the *Twist2*-cre:*Twist1cKO/cKO* embryos (B) compared to littermate controls (A). A and B are low-magnification views of the eye and orbit, demonstrating the disorganized muscles in B. A’ and B’ show high magnification images of individual EOMs, with individual channels displayed to the right. Scale bar in B equals 200 um for A and B; scale bar in B’ equals 50um for all other panels. n = 6 for each genotype.

The transcription factor PITX2 plays an important role in EOM differentiation and maintenance (Diehl et al., 2006; Zhou et al., 2009; Zhou et al., 2011), and loss of *Twist1* under Mesp1-cre leads to loss of *Pitx2* expression (Bildsoe et al., 2013). To determine if the disorganization of the EOMs seen with loss of *Twist1* under *Twist2*-cre is mediated by PITX2, we examined PITX2 expression in *Twist2*:cre:*Twist1*^*cKO/cKO*^ embryos. MYOD is a transcription factor downstream of PITX2 that is required for myogenic differentiation. PITX2 and MYOD expression are similar in EOMs of the mutant and control mice at E13.5 (Figure 2), a developmental time at which disorganization of the EOMs is already observed, indicating that there are additional factors downstream of TWIST1 that influence EOM organization.

### *Twist1* expression is required for the proper formation of EOM tendons

**Figure 3.**
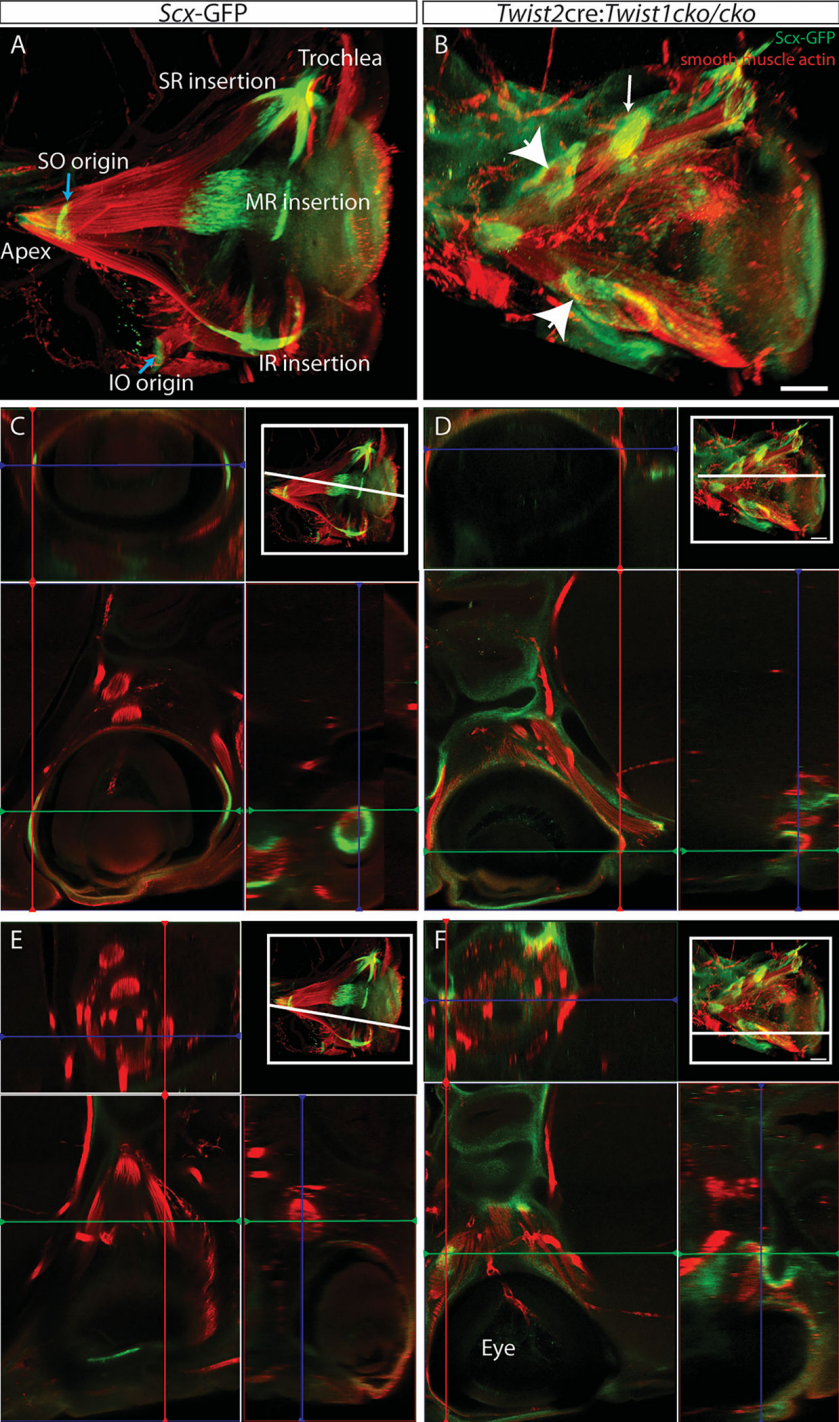
The disorganized EOMs have poorly organized tendons. Heads from E15.5 embryos expressing GFP from the *scx* promoter were whole mounted, stained with anti-GFP (green) and anti-smooth muscle actin (red) antibodies, cleared, and imaged coronally on a confocal microscope. Max intensity projections are shown in A and B, orthogonal views in C-F. In control embryos (A,C,E), tendons are at the apex, scleral insertion of each muscle, origin of the SO and IO muscles (blue arrows) and at the trochlea. In the disorganized EOMs caused by loss of *Twist1* in *Twist2* expressing cells (B,D,F), there are no discrete identifiable tendons inserting on the eyeball. There is a condensation of *scx*-expressing cells at the end of a muscle (white arrow) in the middle of the orbit, and there is diffuse expression of *scx* (white arrowheads) along the length of some muscles. The orthogonal views in C-F are at the level indicated in the upper right inset of each panel. The lower left shows a single Z-plane, with orthogonal views along the red and green lines to the right and above, respectively. C and D are at the same level, through the insertion of the LR and MR in the control. It can be seen in D that there are no discreet muscle insertions with tendons. F is of the area of the lower white arrowhead in B, and E is an equivalent plane in the control. Scale bar equals 200um. For A and B, images were reconstructed in Arivis software and the arteries were cropped out to facilitate visualization. n=4 for each genotype.

The insertion of EOMs onto their appropriate scleral target requires coordinated development of EOM tendons. Thus, to examine the tendons, origins, and scleral insertions of the disorganized muscles, we used *Scx*-GFP reporter mice, which express GFP in tendons throughout the body (Pryce et al., 2007). In the periocular region of E15.5 control mice, GFP signal is discretely concentrated at the origin of the EOMs (annulus of Zinn) and at the points of insertion on the sclera (Figure 3A,C,E). By contrast, the disorganized muscles of E15.5 *Twist2*cre:*Twist1*^*cKO/cKO*^ embryos do not have identifiable tendons inserting on sclera (Figure 3B, D). There are areas of GFP expression along the belly of some EOMs (white arrowheads in 3B, 3F). In some cases, there appears to be a tendon condensation at the end of a disorganized muscle, but it is not inserting on the sclera (white arrow in 3B, see 3F).

### *Twist1* expression is not required in tendon precursors for normal EOM or tendon development

**Figure 4.**
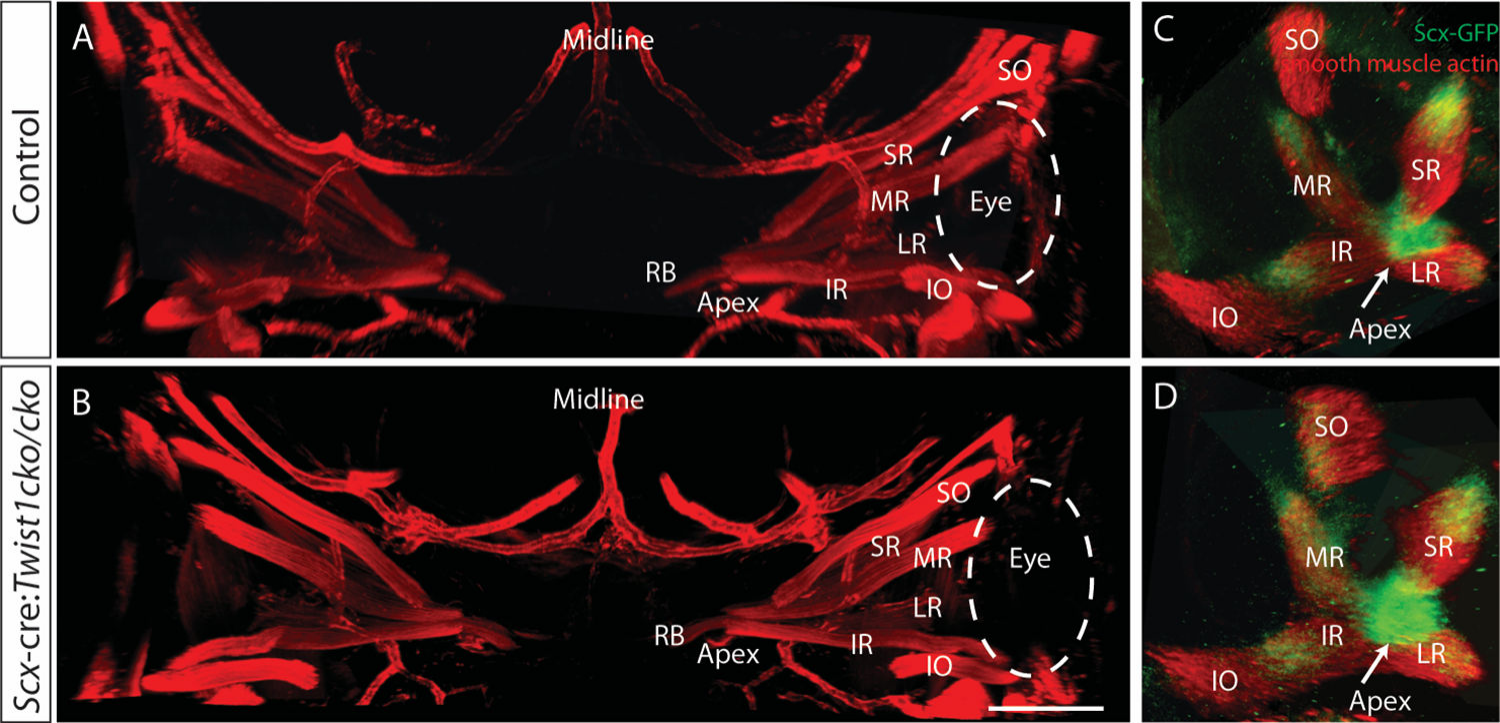
Loss of *Twist1* in *Scx* expressing cells does not disrupt EOM organization or tendon formation. Thick sections from E14.5 embryos stained with anti-smooth muscle actin (red) and imaged coronally show the normal arrangement of the EOMs in both control embryos (A) and *Scx*-cre:*Twist1*^*cKO/cKO*^ embryos (B). Orbits from whole mounts of E13.5 embryos expressing GFP from the scleraxis promoter show normal tendon formation at the apex (arrow) and end of each muscle in both control embryos (C) and *Scx*-cre:*Twist1*^*cKO/cKO*^ embryos (D). Images in C and D were reconstructed in Arivis and are oriented looking into the orbit towards the apex. Abbreviations as per Figure 1. n= 5 Scx-cre+, 3 littermate controls. Scale bar in B equals 100um.

Since the tendons do not form properly in the disorganized EOMs, we next asked whether *Twist1* expression in the tendon precursor cells is necessary for proper EOM and tendon development. *Scleraxis*-cre (*Scx*-cre)(Blitz et al., 2009) is expressed in tendon precursor cells of the EOMs by E11.5 (Comai et al., 2020). In limb muscles, *Scx*-cre also marks a small subset of muscle cells, concentrated near the tendon (Esteves de Lima et al., 2021). We have noted sparse labeling of both mesenchymal and neuroectodermal tissue with *Scx*-cre as early as E9.5 (Supplemental Figure 2). When *Twist1* is knocked out under the control of *Scx*-cre, EOMs develop normally, with normal final patterning (Figure 4). Quantification of the EOM volume showed no significant differences between *Scx*-cre:*Twist1*^*cko/cko*^ embryos and littermate controls who either do not express cre or have a wild-type *Twist1* allele (E14.5: 22.0 +/− 3.3 vs 25.1 +/− 3.8, p=0.17). The tendons also form normally, as evidenced by *Scx*-GFP expression at the origin and insertion of each EOM (Figure 4C–D). Therefore, the abnormal EOM and tendon organization seen with mesenchymal loss of *Twist1* is not secondary to loss of *Twist1* in the tendon precursors. Additionally, tendon formation is not dependent on *Twist1* expression in tendon precursor cells and the tendon abnormalities seen in *Twist2*-cre:*Twist1*^*cko/cko*^ animals are secondary to abnormal EOM development.

### Abnormal configuration of the EOMs leads to abnormal branching of the cranial motor neurons

**Figure 5.**
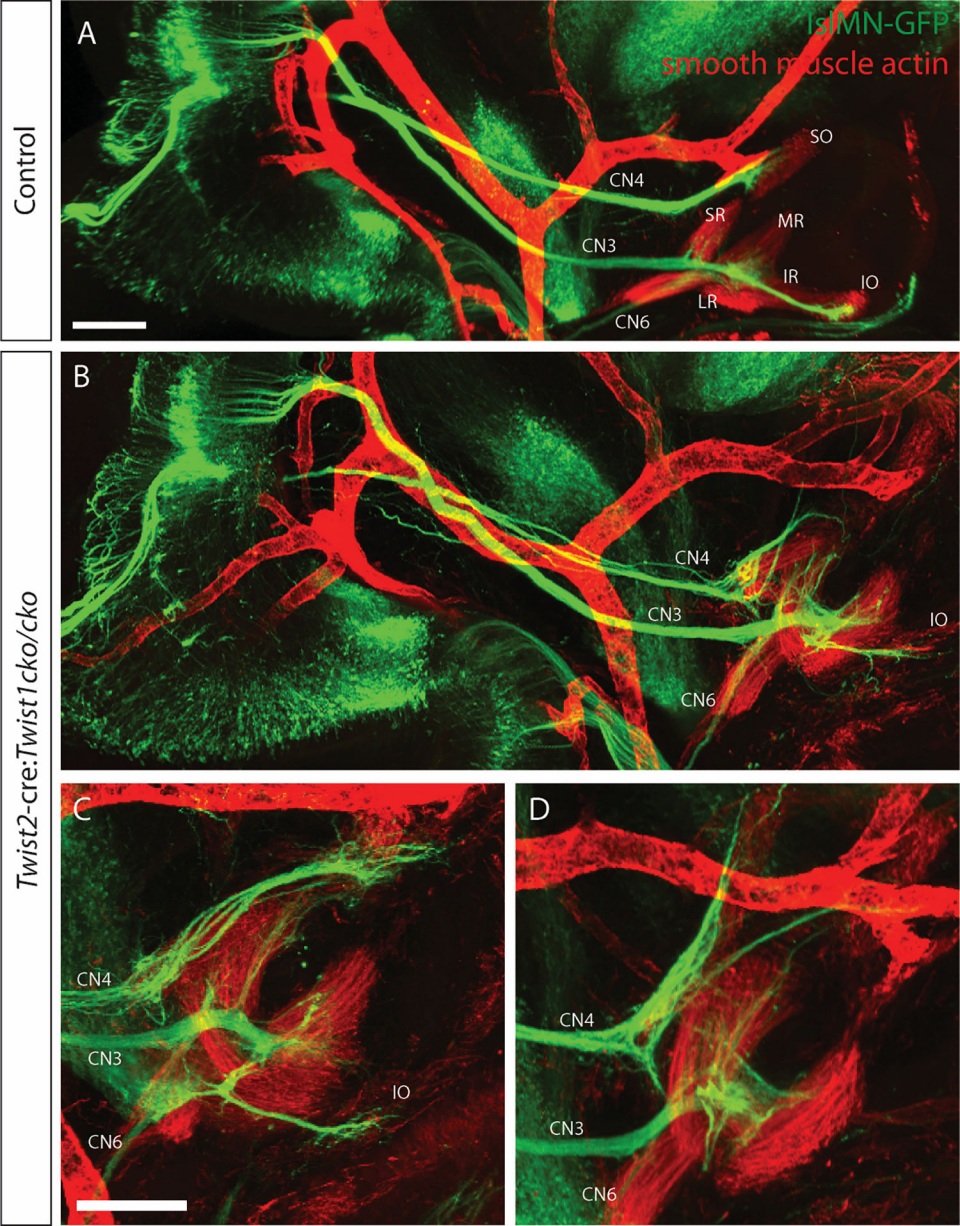
Disorganization of the EOMs leads to abnormal branching of the innervating cranial motor neurons. E13.5 embryos expressing GFP from the *Isl1*^MN^ promoter were whole mounted, stained with anti-GFP (green) and anti-smooth muscle actin (red, labels muscles and arteries) antibodies, cleared, and imaged sagittally on a confocal microscope. In a control embryo (A) CN4 innervates the SO muscle, CN3 innervates the MR, IR, IO and SR, and CN6 innervates the LR. In *Twist2*-cre:*Twist1*^*cKO/cKO*^ embryos (B-D, examples of three different embryos), the EOMs are disorganized and the ocular motor nerves have abnormal branching patterns. CN4 is defasciculated and sends aberrant branches to the superior aspect of the orbit, as well as to muscle fibers that may be SO. CN3 enters the orbit in the correct location but then extends exuberant aberrant branches in addition to normal branches, and CN6 enters the orbit but cannot be visualized innervating any muscle. Scale bar in A equals 300um in A and B. Scale bar in C equals 300um in C and D. Abbreviations as in Figure 1. n=6 of each genotype.

The EOMs are innervated by the axons of oculomotor (CN3), trochlear (CN4), and abducens (CN6) cranial motor neurons that develop as the muscles are developing. At E10.5, axons have exited the motor nuclei and begun growing towards the orbit. A few pioneer axons contact PITX2+ cells ventrolateral to the eye as early as E10.0 (Bjorke et al., 2021). The majority of axons reach the orbit around E11.5, as the muscle anlage is differentiating (Bjorke et al., 2021; Michalak et al., 2017). Normally, CN3 travels ventrally from the oculomotor nucleus in the midbrain, through the region that will become the cavernous sinus, and enters the orbit. The superior division branches off from the main oculomotor trunk to innervate the superior rectus and levator palpabrae superioris muscles. The main trunk becomes the inferior branch and forms a decision region, which sends short branches to the medial rectus and inferior rectus muscles and a long branch to the inferior oblique muscle. CN4 exits the midbrain dorsally, crosses the midline, then courses ventrally to the opposite orbit, where it innervates the superior oblique muscle. CN6 travels from its origin in the hindbrain, along the retractor bulbi, to innervate the lateral rectus muscle (Figure 5A). We have previously shown that in the absence of the EOMs, the cranial nerves target the orbit correctly, but fail to make terminal branches (Michalak et al., 2017). In the presence of disorganized EOMs in the *Twist2*-cre:*Twist1*^*cko/cko*^, the cranial nerves also target the orbit correctly, but then branch abnormally (Figure 5B–D). CN4 is defasciculated and sends multiple branches to the superior aspect of the orbit, some of which do not appear to be contacting muscle tissue. CN3 forms a large decision region near the origin of the EOMs from which multiple aberrant branches extend. In some embryos, a branch resembling the wildtype branch to the inferior oblique forms, but exits the inferior decision region at an abnormal angle and appears to make two right-angle turns to course aberrantly towards the anterior orbit (Figure 5B–C). CN6 can be seen coursing along the retractor bulbi muscle (which appears the most normal and identifiable of the EOMs), but does not contact any muscles in the orbit. These observations suggest that appropriate EOM organization is required for the terminal branching and insertion of cranial motor neurons.

## Discussion

The EOMs are a unique muscle group based on their molecular composition and function (Porter, 2002). EOM and orbital development is also unique because tissues are derived from different embryological origins. Orbital connective tissue at the level of the EOM insertions is derived from neural crest cells (NCCs), while the connective tissue at the level of the EOM origin at the annulus of Zinn originates from the cranial mesoderm (Comai et al., 2020). Although much is known about the myogenic transcription factors required for initial EOM differentiation, little is known about how the distinct muscles form and project from the initial anlage of muscle precursors. Here, we show that mesenchymal expression of the transcription factor TWIST1 is necessary for the proper organization of EOMs into distinct muscles and formation of normal tendon insertions onto the sclera.

Knock-out of *Twist1* under either *Pdgfrb*-cre or *Twist2*-cre leads to disorganization of the EOMs, of varying severity. Both *Pdgfrb*-cre and *Twist2*-cre are expressed in cranial mesenchyme of both mesodermal and cranial neural crest origin. *Twist2* expression begins as early as E8.5 and is relatively enriched in the cranial mesoderm versus the cranial neural crest, although it is expressed in both (Bildsoe et al., 2016). *Pdgfrb* is expressed in mesenchyme throughout the body as early as E10.5 (Shinbrot et al., 1994). The conditional loss of *Twist1* under these cre-recombinase-expressing lines allows for the interrogation of *Twist1*’s role in EOM development distinct from its role in neural tube closure, unlike *Mesp1*-cre. The disorganization of the EOMs we show here with these conditional knockouts of *Twist1* reveals that *Twist1* expression is necessary for proper differentiation of the EOMs into distinct muscles. The *Twist2*:cre mutants also displayed a larger total EOM volume. This could be the result of disruption of the normal control of muscle precursor division, leading to overproduction of muscle cells, or could indicate that the process of individual muscle separation/differentiation also influences muscle size.

We hypothesized that the effects of loss of *Twist1* may be mediated by *Pitx2* for several reasons: *Pitx2* is required for EOM development (Gage et al., 1999); *Pitx2* expression is regulated by retinoic acid (Bohnsack et al., 2012; Kumar and Duester, 2010); and blockade of retinoic acid signaling, either genetically or pharmacologically during early development of the initial EOM anlage, also leads to abnormal EOM patterning (Bohnsack et al., 2012; Comai et al., 2020). PITX2 expression was not altered in the mutant muscles, however, nor was its downstream target MYOD, indicating both that PITX2 and MYOD expression are not sufficient for normal EOM differentiation and that *Twist1* is acting through an alternate pathway to control EOM patterning.

In addition to the disorganization of the EOMs, we also show that tendons do not form properly in *Twist2*-cre:*Twist1*^*cko/cko*^ mice. Tendons form from *Scx*-expressing precursors (Murchison et al., 2007; Schweitzer et al., 2001). In the limbs, the tendons attach muscle to bone; bone superstructures provide anchoring points for tendons. These bony superstructures initially form independent of the muscle from precursors that express *Scx* and *Sox9* (Blitz et al., 2009; Sugimoto et al., 2013); later maintenance and growth of these bony structures requires signals from both tendon and muscle (Huang, 2017). Unlike limb muscles, however, EOMs insert onto sclera of the eyeball rather than onto bone and there are no bony superstructures for the tendons to insert onto. Analogous to development of the bony superstructures, *Scx* and *Sox9* are also expressed in the periocular mesenchyme, initially broadly and then restricted to the tendon insertion sites by E13.5 (Comai et al., 2020). In *Twist2*-cre:*Twist1*^*cko/cko*^ mice, instead of distinct, *Scx*-expressing tendons at the origin and insertion of each EOM, mutant animals have *Scx*-expressing cells in clumps throughout the EOMs. Some clumps do appear to be at the end of a muscle, but not attached to sclera. *Scx*-GFP has been reported to also label muscle connective tissue (Comai et al., 2020) and some myogenic cells come from a *Scx* lineage (Esteves de Lima et al., 2021), so this ectopic GFP expression may represent abnormal muscle or connective tissue differentiation in the mutant animals, with resulting abnormal expression of scleraxis. To determine the role of *Twist1* expression in the tendon precursor cells, we knocked out *Twist1* under the control of *Scx*-cre, but this did not result in any abnormalities of EOM or tendon formation. These results suggest that the disorganized tendons are not caused by lack of Twist1 expression in the tendon precursor cells, but instead that normal EOM organization is required for proper tendon formation. Thus, the lack of tendon formation is a secondary effect, and tendon formation is dependent on proper EOM formation.

Our results also provide further evidence that EOM identity influences axon guidance decisions of the innervating cranial motor neurons. In the presence of disorganized muscles, the axon decision regions also appear disorganized. Neither *Twist1* nor *Twist2-cre* are expressed in motor neurons, so these neuronal changes must be secondary to the muscle changes. In the complete absence of EOMs, motor neurons target the orbit correctly, but then fail to make correct terminal branches and many later die (Bjorke et al., 2021; Michalak et al., 2017). The exact branching errors depend on the genetic strategy used to ablate the EOMs. With the *Myf5* cre/cre allele, which disrupts both *Myf5* and *Mrf4*, axons fail to make terminal branches and many die (Michalak et al., 2017). In *Myf5*-DTA mutants, in which *Myf5* expressing cells are killed, some embryos have a similar lack of branches and some form ectopic branches (Bjorke et al., 2021). By contrast, *Pitx2*^*−/−*^ embryos, which disrupt an earlier stage of muscle development, show more severe axon errors, with a failure to stop near the eye and long, misdirected branches (Bjorke et al., 2021). Here, we find that in the presence of disorganized muscles, instead of failing to make branches, the motor neurons make aberrant branches, and often these branches do not appear to be contacting any muscle. In this way, they are most similar to the *Pitx2* knockouts. The EOMs in the *Twist* mutants are not just spatially disorganized, but are also missing key elements of EOM differentiation. Thus, these findings suggest that individual EOMs express or secrete cues that guide the appropriate incoming axons to them, and that such expression is disrupted by loss of *Twist1* or the resulting disorganization and lack of individual muscle identity. The identity of the cues that lead incoming axons to the proper EOM are not currently known.

We have shown that mesenchymal expression of *Twist1* is necessary for the proper separation of the EOM muscle anlage into distinct EOMs. Secondarily, properly formed EOMs are required for appropriate tendon formation and for axon guidance of the cranial motor nerves. Strabismus in patients with *TWIST1* variants, therefore, may be caused by abnormal development of the EOMs and resulting abnormalities in tendon formation and innervation.

## Supplementary Material

1 Supplemental Figure 1: *Twist2*-cre expression. Coronal images from E9.5 *Twist2*-cre:TdTomato fluorescent reporter embryos shows sparse labeling in the mesenchyme surrounding the optic vesicle (A, B, D) and more robust labeling in more posterior mesenchyme (B,C). There is no labeling in neuronal tissues. Scale bar in C equals 200um in A-C. Scale bar in D equals 50um.

2 Supplemental Figure 2: *Scx*-cre expression. Coronal images from E9.5 *Scx*-cre:TdTomato fluorescent reporter embryos shows sparse labeling in the mesenchyme (A-C). Note the labeling in some neuronal tissues. Scale bar in C equals 200um in A-C.

## Highlights

- Loss of TWIST1 in cranial mesenchyme leads to disorganized extraocular muscles
- Disorganized extraocular muscles have abnormal tendon formation
- TWIST1 is not required in tendon precursors for normal extraocular muscle and tendon development
- Cranial motor neurons make abnormal branches in the presence of disorganized muscles
