# Supplementary figures and images for: TWIST1, a gene associated with Saethre-Chotzen syndrome, regulates extraocular muscle organization in mouse
Source: Dev Biol. Author manuscript; Available in PMC 2022 Dec 20. (PMC9765759; doi:10.1016/j.ydbio.2022.07.010)

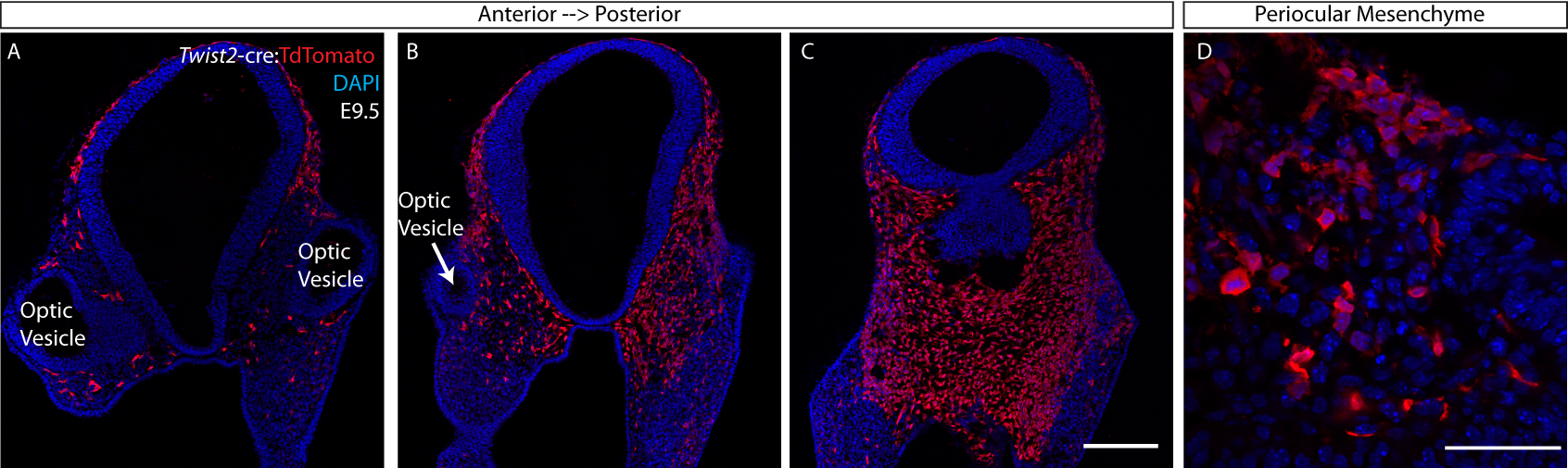

Supplement: 1 — Supplemental Figure 1: Twist2-cre expression. Coronal images from E9.5 Twist2-cre:TdTomato fluorescent reporter embryos shows sparse labeling in the mesenchyme surrounding the optic vesicle (A, B, D) and more robust labeling in more posterior mesenchyme (B,C). There is no labeling in neuronal tissues. Scale bar in C equals 200um in A-C. Scale bar in D equals 50um. [file NIHMS1851786-supplement-1.tif]

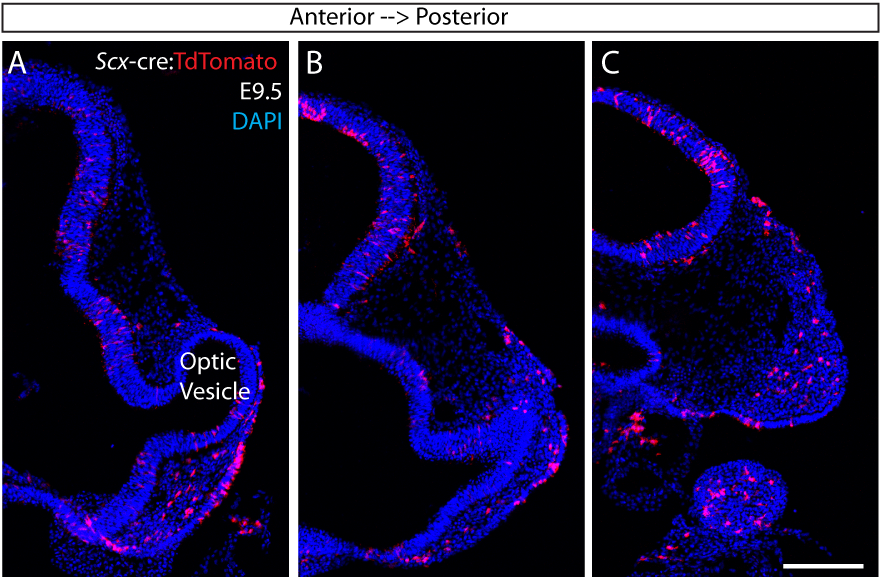

Supplement: 2 — Supplemental Figure 2: Scx-cre expression. Coronal images from E9.5 Scx-cre:TdTomato fluorescent reporter embryos shows sparse labeling in the mesenchyme (A-C). Note the labeling in some neuronal tissues. Scale bar in C equals 200um in A-C. [file NIHMS1851786-supplement-2.tif]
